# Enhanced mechanical, thermal, and electric properties of graphene aerogels via supercritical ethanol drying and high-temperature thermal reduction

**DOI:** 10.1038/s41598-017-01601-x

**Published:** 2017-05-03

**Authors:** Yehong Cheng, Shanbao Zhou, Ping Hu, Guangdong Zhao, Yongxia Li, Xinghong Zhang, Wenbo Han

**Affiliations:** Science and Technology on Advanced Composites in Special Environment Laboratory, Harbin Institute of Technology, Harbin, 150080 People’s Republic of China

## Abstract

Graphene aerogels with high surface areas, ultra-low densities and thermal conductivities have been prepared to exploit their wide applications from pollution adsorption to energy storage, supercapacitor, and thermal insulation. However, the low mechanical properties, poor thermal stability and electric conductivity restrict these aerogels’ applications. In this paper, we prepared mechanically strong graphene aerogels with large BET surface areas, low thermal conductivities, high thermal stability and electric conductivities via hydrothermal reduction and supercritical ethanol drying. Annealing at 1500 °C resulted in slightly increased thermal conductivity and further improvement in mechanical properties, oxidation temperature and electric conductivity of the graphene aerogel. The large BET surface areas, together with strong mechanical properties, low thermal conductivities, high thermal stability and electrical conductivities made these graphene aerogels feasible candidates for use in a number of fields covering from batteries to sensors, electrodes, lightweight conductor and insulation materials.

## Introduction

Inorganic^1, 2^, carbon^3, 4^, and organic aerogels^5^ with high porosities and ultra-light weights have been prepared to exploit their wide applications from pollution adsorption to energy storage, catalyst supports, supercapacitor, and thermal insulation^1–5^. Compared with those aerogels, graphene aerogels possess larger Brunauer-Emmett-Teller (BET) surface areas (S_BET_ > 500 m^2^∙g^−1^)^6–8^ and ultra-low densities^9, 10^ because of the very high S_BET_ of a single graphene sheet (theoretical value of 2600 m^2^∙g^−1^)^11^. Tang *et al*. synthesized ultra-light graphene aerogels with densities from 1.8 mg∙cm^−3^ to 27.2 mg∙cm^−3^ using different starting graphene oxide (GO) concentrations. Despite the good thermal conductivity of single-layer graphene (4.84 × 10^3^–5.30 × 10^3^ W·m^−1^·K^−1^)^12^, the nanoporous structure, low density, and perfect opacity of graphene aerogel depress the gaseous thermal conductivity^13^, solid thermal conductivity^14^, and radiant thermal conductivity^10^, respectively. Moreover, Yue *et al*.^10^ demonstrated the defects in graphene, and the relatively small sizes of the graphene sheets further suppressed thermal conductivity. Due to the larger thermal contact resistance at the interfaces between adjacent GO sheets^15^, Xie *et al*. prepared graphene aerogels with ultra-low thermal conductivities (as low as 4.7 × 10^−3^–5.9 × 10^−3^ W·m^−1^·K^−1^) at room temperature approximately 80% lower than that of air (0.0257 W·m^−1^·K^−1^ at 20 °C).

Taking advantage of larger surface areas or lower thermal conductivities, current researches on graphene aerogels mainly focused on the ultra-light weight^16, 17^, highly compressibility (strain>50%)^16, 17^, recoverability^18^ and thermal insulation^10^. The poor mechanical, thermal, and electric properties restrict their applications in energy storage, catalyst supports, supercapacitor, and thermal insulation. Ultralight and ultra-flyweight graphene aerogels have highly compressibility and recoverability at maximum strain ≥50%, however, the compression strengths were in the order of KPa^16, 17, 19^. Several studies have promoted the mechanical properties of graphene aerogels. Zhang *et al*.^7^ created mechanically strong graphene aerogels which were dried by supercritical CO_2_ and with L-ascorbic acid as the reducing agent. The yield strengths and Young’s moduli of these aerogels ranged from 0.04 MPa to 0.66 MPa and from 1.2 MPa to 6.2 MPa, respectively, with densities in the range of 12–96 mg∙cm^−3^. Huang *et al*.^20^ have prepared PEI-crosslinked graphene aerogel with yield strength and Young’s modulus of 1 MPa and 20 MPa. The thermal stability of graphene aerogels was depended on reduction level. High-temperature annealing of graphene aerogels results in enhanced thermal stability^21, 22^ and an increase in Young’s moduli by one order of magnitude^22^. Graphene aerogel annealed at 400 °C exhibits significantly improved thermally stability compared with GO and as-prepared graphene aerogel^21^. With annealing temperature increases from 1050 °C to 2500 °C, the graphene aerogels become more resistant to oxidation in air and exhibit an approximately 200 °C improvement in oxidation temperature^22^. The bulk electrical conductivity of macroscopic 3D graphene networks was less than 1 S/m^23, 24^. The electrical conductivity of graphene aerogels increased to >550 S/m after annealing at 2500 °C^22^. Chen *et al*.^21^ have studied the effect of various reducing agents on the electrical conductivities of the graphene aerogels and found that the electrical conductivity of the hydrogel prepared using HI and NaHSO_3_ have higher values.

In this paper, we prepared mechanically strong graphene aerogels with large BET surface areas, low thermal conductivities, high thermal stability and electric conductivities via hydrothermal reduction and supercritical ethanol drying. Annealing at 1500 °C resulted in slightly increased thermal conductivity and further improvement in mechanical properties, oxidation temperature and electric conductivity of the graphene aerogel. The large BET surface areas, together with strong mechanical properties, low thermal conductivities, high thermal stability and electrical conductivities made these graphene aerogels feasible candidates for use in a number of fields covering from batteries to sensors, electrodes, lightweight conductor and insulation materials.

## Results and Discussion

### Synthesis of the aerogels

**Figure 1:**
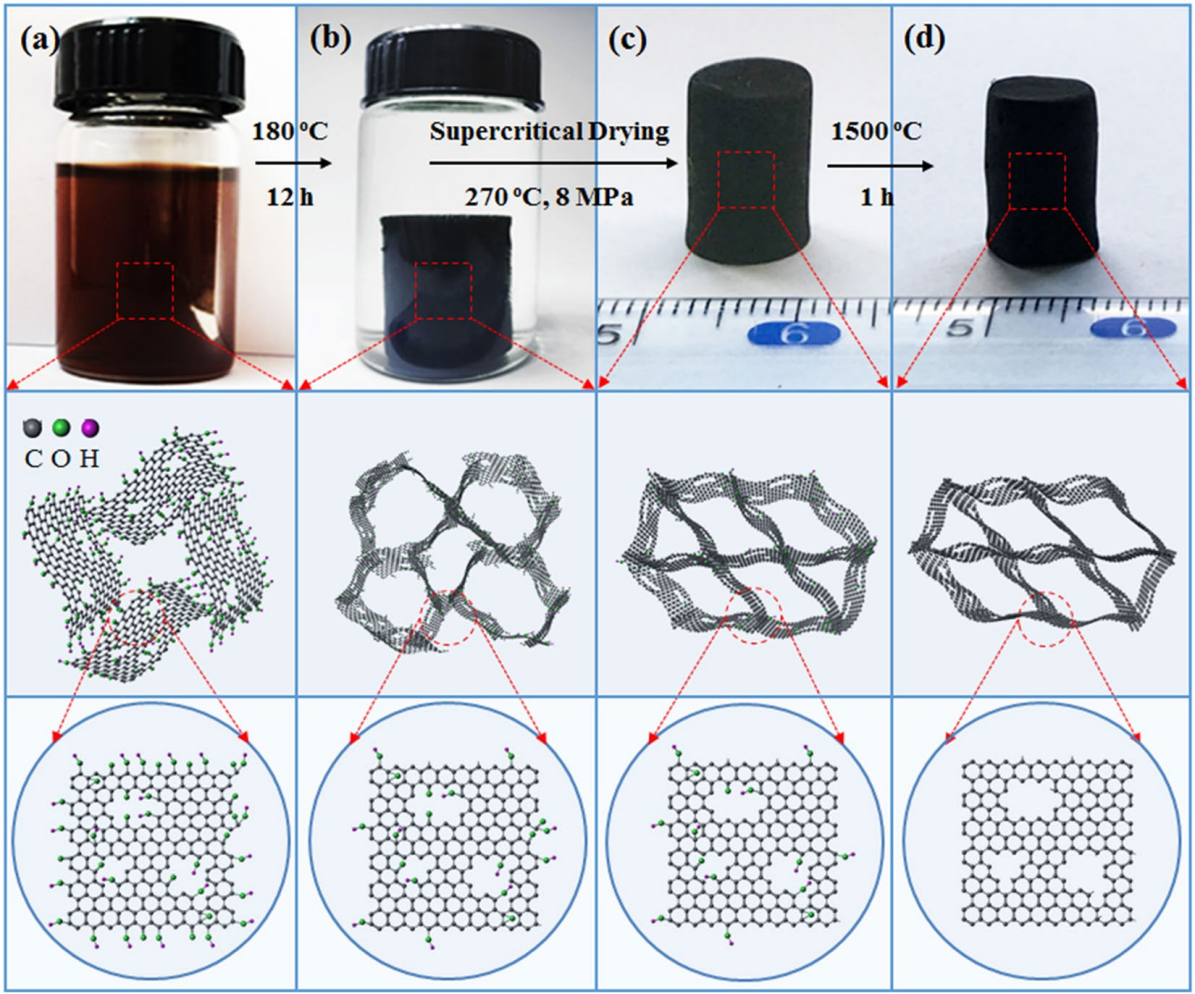
Synthesis of graphene aerogels. (**a**) GO aqueous dispersion, (**b**) graphene hydrogel, (**c**) dried graphene aerogels and (**d**) annealed graphene aerogels.

The synthesis of the mechanically strong graphene aerogels with cylindrical morphology is illustrated in Fig. 1. GO aqueous dispersion prepared from natural graphite using the improved Hummers method^25^. Graphene hydrogels were prepared by hydrothermal reduction of GO dispersions with the assistance of a mild reducing agent NaHSO_3_. The obtained gels were dried using supercritical ethanol to obtain the graphene aerogels, the resulting samples were referred to as GA-S. The obtained GA-S samples were then heat treated at 1500 °C, the resulting samples were labeled as GA-S-1500C. The GA-S and GA-S-1500C prepared with various concentrations of GO (C_GO_) were denoted as GA-Sx and GA-Sx-1500C, where x is the concentration of GO dispersion in milligram per milliliter. In addition, we investigated the effect of the drying process by freeze-drying graphene aerogels with GO aqueous dispersion of 5 mg∙mL^−1^ using the same method as that for GA-S, but deionized water, instead of ethanol, was used to purify the hydrogels. The resulting sample were denoted as GA-F.

### Characterization of materials

The removal of a large amount of water from the highly porous network interconnected by partial overlapping or coalescing flexible graphene sheets resulted in the formation of the 3D framework. The graphene aerogels exhibited an interconnecting porous structure of randomly oriented graphene sheets with wrinkled texture. The obtained hydrogels had smaller pore sizes, because of remarkable shrinkage in the volume of GO dispersion during hydrothermal reduction process. The poor microscopic structure of GA-F (Fig. S2) was mainly attributed to the volume expansion of liquid water in the networks of hydrogels generated in the process of pre-frozen into ice which destroyed the original fine framework of the graphene hydrogels. The collapse of the cross-linking network during subsequent sublimation process generated the poor microscopic structure of GA-F which was rich in highly unordered interconnected macro pores.

**Figure 2:**
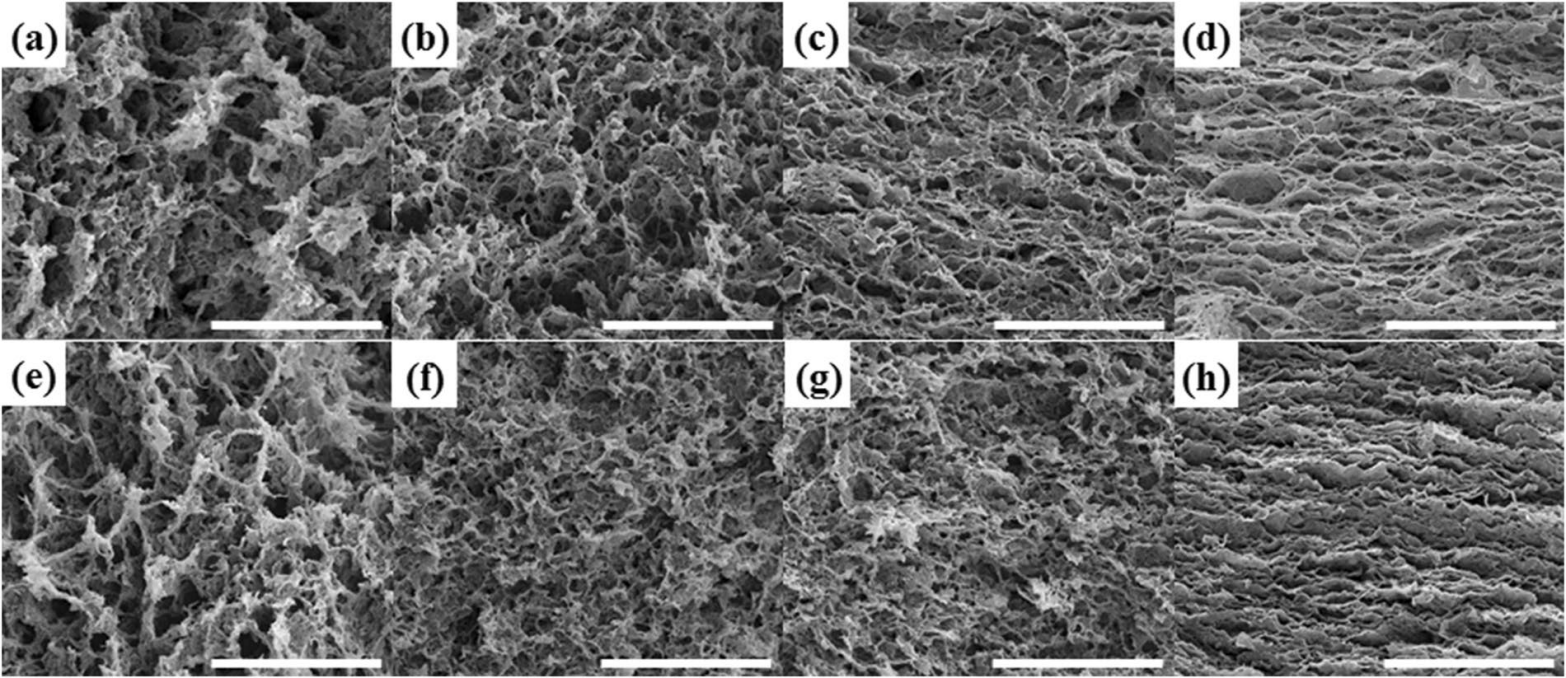
Microscopic morphology of the graphene aerogels prepared by hydrothermal reduction of graphene oxide (GO) dispersions with different concentrations (**a–d**) before and (**e** and **f**) after heat-treatment at 1500 °C under N_2_ atmosphere for 1 h. C_GO_ values of (**a** and **e**) 1, (**b** and **f**) 2, (**c** and **g**) 5, and (**d,h**) 10 mg∙mL^−1^. The scale bar is 10 µm.

Supercritical drying methods include high temperature supercritical drying from solvents such as methanol, ethanol and acetone, and low temperature supercritical drying from carbon dioxide^26^. They both circumvent the destructive differential capillary stresses of conventional evaporative drying either by transferring the solvent into its supercritical state or by replacing the solvent mainly with supercritical CO_2_, thus suppressing any liquid-vapor interface inside the sol-gel product^27^. The usually used ethanol was chosen as supercritical drying solvent in our experiments. When ethanol in autoclave was forced to a temperature and pressure above its critical point (243.1 °C, 6.38 MPa)^28^, it transformed into a supercritical fluid. Above the critical point, the various properties of supercritical ethanol are between those of gas and liquid. Although the density of supercritical fluid is similar to that of liquid, the viscosity is similar to that of gas, and its diffusivity is between the two states^29^. Because the supercritical fluid has lower viscosity and higher diffusion velocity than liquid, it can easily diffuse through the pores of the gel, resulting in higher drying efficiency. Shrinkage of a gel during drying is driven by the capillary pressure, which can be reduced by reducing surface tension of the pore liquid, and supercritical drying eliminates surface tension and keeps the structure of original fine framework of the graphene gels from destroying to obtain aerogels^30^. Drying process impacted the microstructure of aerogels, and further affected the physical properties of aerogels, such as bulk density, mechanical property, thermal and electrical conductivity. The microscopic morphology of GA-S and GA-S-1500C graphene aerogels prepared with 1, 2, 5, and 10 mg∙mL^−1^ GO are shown in Fig. 2. 3D architectures of GA-S and GA-S-1500C showed homogeneously interconnected pores with sizes of less than 2 µm. With an increase in C_GO_ from 1 mg∙mL^−1^ to 10 mg∙mL^−1^ or after high-temperature annealing, the pore structure of graphene aerogels appeared to be much finer and ordered, so, the network architecture, especially those of GA-S10 and GA-S10-1500C, became much “stronger” to support greater external load. The scanning images of graphene aerogels prepared by supercritical drying and freezing drying demonstrated supercritical dried graphene aerogels had more fine, uniform and ordered microscopic morphology. Digital photographs of the GA-S and GA-S-1500C samples are shown in Figs S3 and S4, respectively.

**Figure 3:**
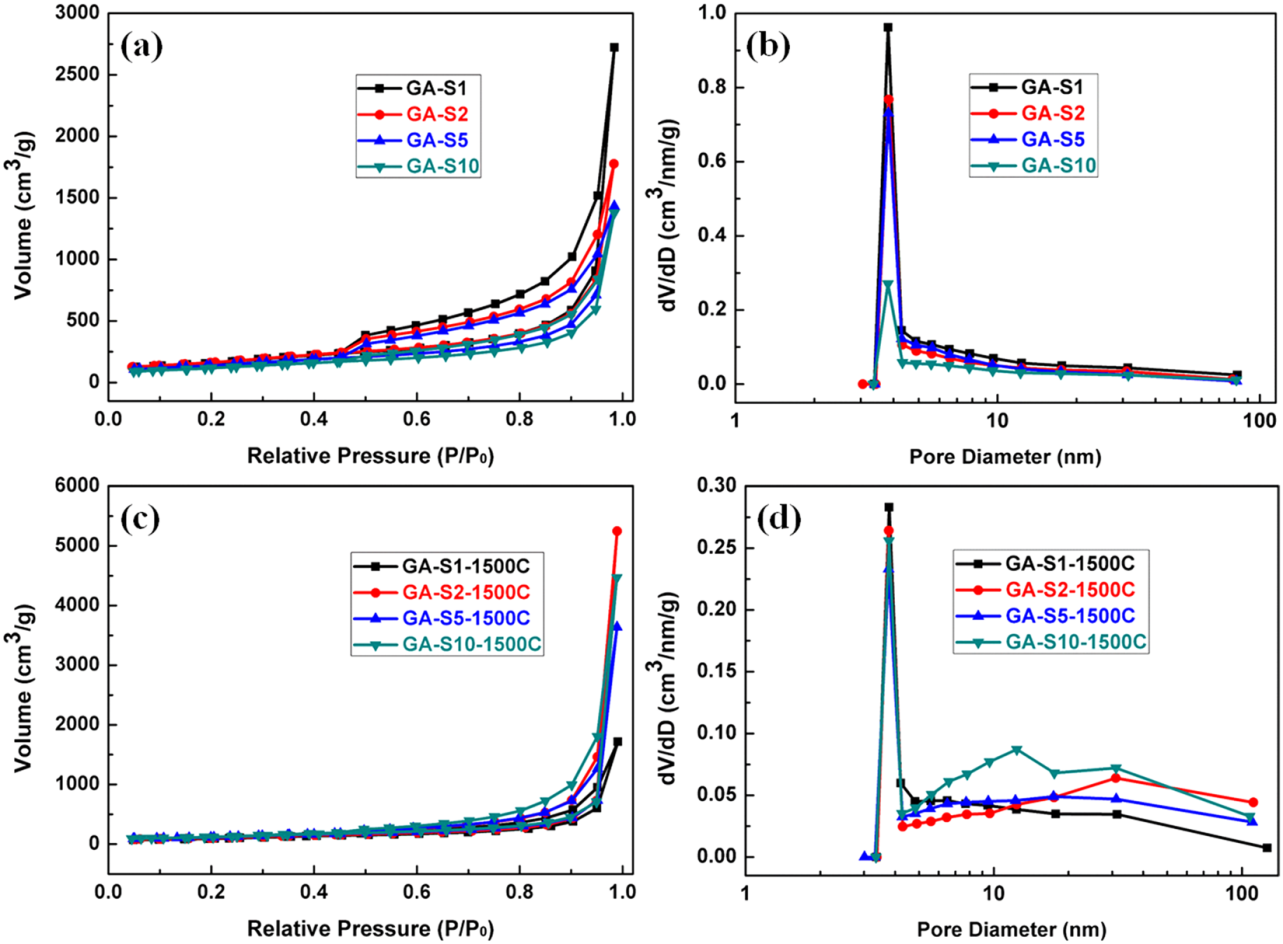
Nitrogen adsorption–desorption isotherms and Barrete-Joynere-Halenda (BJH) desorption pore distribution plots of (**a** and **b**) GA-S and (**c** and **d**) GA-S-1500C.

Except for the macropores observed in the electron micrographs, the mesoporous feature of graphene aerogels was investigated by nitrogen adsorption–desorption measurements. The type IV adsorption–desorption isotherms shown in Fig. 3a and c for all GA-S and GA-S-1500C samples indicate that significant mesopores existed in the frameworks. The isotherms of GA-S and GA-S-1500C exhibited an H_3_-type hysteresis loop at high relative pressure, which was typical characteristic for aggregates of plate-like particles and open large pores^31^. The total adsorption volumes and the size of the hysteresis loops of the GA-S samples decreased as C_GO_ increased. Moreover, the pore size distribution of GA-S and GA-S-1500C calculated by the BJH method indicated a large proportion of mesopores in a narrow distribution from 3 nm to 5 nm with peak pore diameter of approximately 3.8 nm (Fig. 3b and d). Annealing of GA-S samples at 1500 °C reduced the proportion of pore diameter in the range of 3–5 nm. Thus, the relative quantity of pore diameter exceeding 5 nm correspondingly increased.

The surface areas (S_BET_), pore volumes, and average pore diameters of GA-S and GA-S-1500C were shown in Table S1. The S_BET_ of the graphene aerogels were much lower than 2600 m^2^∙g^−1^ of the theoretical value for a single graphene sheet^11^ because of the layering or overlapping of graphene sheets^8^. This values apparently improved by reducing C_GO_ or enhancing the exfoliation of graphene sheets during the self-assembling process^6^. The S_BET_ and pore volume of GA-S1 at approximately 600 m^2^∙g^−1^ and 4.487 cm^3^∙g^−1^, respectively, decreased to corresponding values of 435.6 m^2^∙g^−1^ and 2.153 m^3^∙g^−1^ for GA-S10 as C_GO_ increased. The dramatic decline in the surface area of the graphene aerogels at high C_GO_ was attributed to the improved sites of partial overlapping or cross-linking of the flexible graphene sheets generated during the self-assembling process to form a 3D network with higher density. The healing or repairing of defects in the graphene sheet reduced the textural mesopores, accounting for the lower S_BET_ of GA-S-1500C than GA-S. However, the S_BET_ of GA-S-1500C increased from 336.5 m^2^∙g^−1^ with 1 mg∙mL^−1^ C_GO_ to 440.8 m^2^∙g^−1^ with 10 mg∙mL^−1^ C_GO_, this trend was also broadly applicable to the desorption surface area cumulated by BJH method (Table S1). This phenomenon could be attributed to the relative amounts of larger textural mesopores with a diameter exceeding 3.8 nm increased along with the increase of C_GO_. As shown in the BJH desorption pore distribution plots of GA-S-1500C (Fig. 3d), the vast majority of mesopores distributed in the main peak at 3.8 nm. When the diameter of pores exceeding 3.8 nm, GA-S-1500C samples prepared by high concentration GO had the larger ordinate values in desorption pore distribution plots, that means the increase of the relative amounts of larger mesopores. The increase of the relative amounts of larger textural mesopores existed in GA-S-1500C samples with high C_GO_ formed such a trend in the surface area.

**Figure 4:**
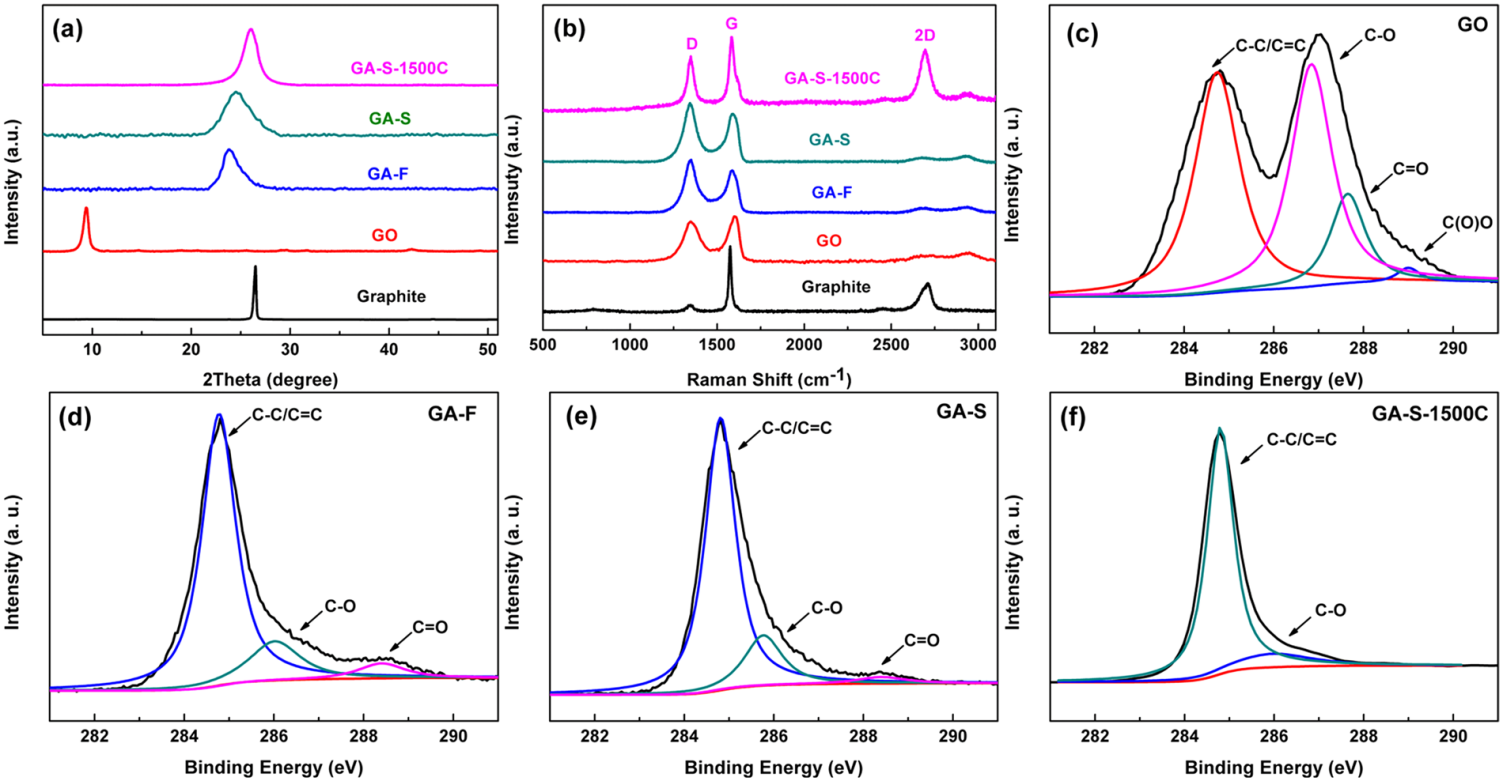
(**a**) X-ray diffraction patterns and (**b**) Raman spectra of graphite, GO, GA-F, GA-S, and GA-S-1500C. The C 1 s peak in the X-ray photoelectron spectra of (**c**) GO, (**d**) GA-F, (**e**) GA-S, and (**f**) GA-S-1500C.

The XRD patterns of GA-F, GA-S, and GA-S-1500C compared with those of GO and graphite are depicted in Fig. 4a. The peak position for graphite powder appeared at 2θ = 26.4°. The typical broad peak of the GO film was observed at 2θ = 9.4°. Compared with the diffraction peak located at 2θ = 23.8° for GA-F, the peak of GA-S showed a slight shift to a higher 2θ angle (24.5°), suggesting that GA-S was well reduced at high-temperature supercritical drying. The value of 2θ increased to approximately 26° for GA-S-1500C, demonstrating that the graphene sheets had a highly crystalline structure after annealing at 1500 °C. The micro-Raman spectra of graphite, GO, GA-F, and GA-S are shown in Fig. 4b. Compared with the G peak of graphite at approximately around 1575 cm^−1^, the shift in the G peak for GO (1599 cm^−1^) was attributed to the presence of isolated double bonds that resonate at frequencies higher than that of the G-band of graphite^32^. The presence of G-bands for GA-F and GA-S at 1586 and 1590 cm^−1^, respectively, were ascribed to the recovery of the hexagonal network of carbon atoms with defects^33^. After hydrothermal reduction, the significant increase of I_D_/I_G_ for GA-F and GA-S indicate a decrease in the average size but an increase in the number of sp2 domains upon reduction of the exfoliated GO^32^. The reduction effect obviously cannot be considered as the healing or repairing of defects in GO^34^. Annealing at 1500 °C resulted in the evident decrease in I_D_/I_G_, strongly suggesting the removal of a considerable number of defects in the graphene sheets. The intensity of the two-dimensional (2D) peak (2695 cm^−1^) for GA-S-1500C increased dramatically, revealing better graphitization of graphene sheets after annealing at 1500 °C^35^.

The C1s X-ray photoelectron spectrum of GO clearly indicated considerable oxidation with four components that corresponded to carbon atoms in four functional groups, namely, C–C/C=C (284.8 eV), C–O (hydroxyl and epoxy, 286.8 eV)^36^, C=O (carbonyl, 287.6 eV), and O–C=O (carboxyl, 289.0 eV)^37^ (Fig. 4c). However, the C1s XPS spectra of GA-F and GA-S (Fig. 4d and e) only exhibited two oxygen functional groups (C–O and C=O), and their peak intensities were much weaker than those in GO. The C1s spectrum of GA-F consisted of three main components located at binding energies of 284.8, 286.0^37^, and 288.3 eV^38^, which were attributed to the C–C/C=C, C–O, and C=O functional groups, respectively. The C1s X-ray photoelectron spectrum of GA-S clearly indicated a considerable degree of reduction with the same three components. However, the binding energies of C–O and C=O peaks of GA-S shifted into 285.8 and 288.4 eV, respectively, and the peak intensity of C=O was much weaker than that of GA-F on account of the higher drying temperature of GA-S samples. The oxygen-containing functional groups were reduced greatly after the graphene aerogels were annealed at 1500 °C. The main peak of C–C/C=C located at 284.8 eV still showed a tail band centered at 286.0 eV (Fig. 4f) corresponding to the C–O bond. Owing to high-temperature thermal reduction, the areas of the C–O peak decreased dramatically.

### Mechanical properties

**Figure 5:**
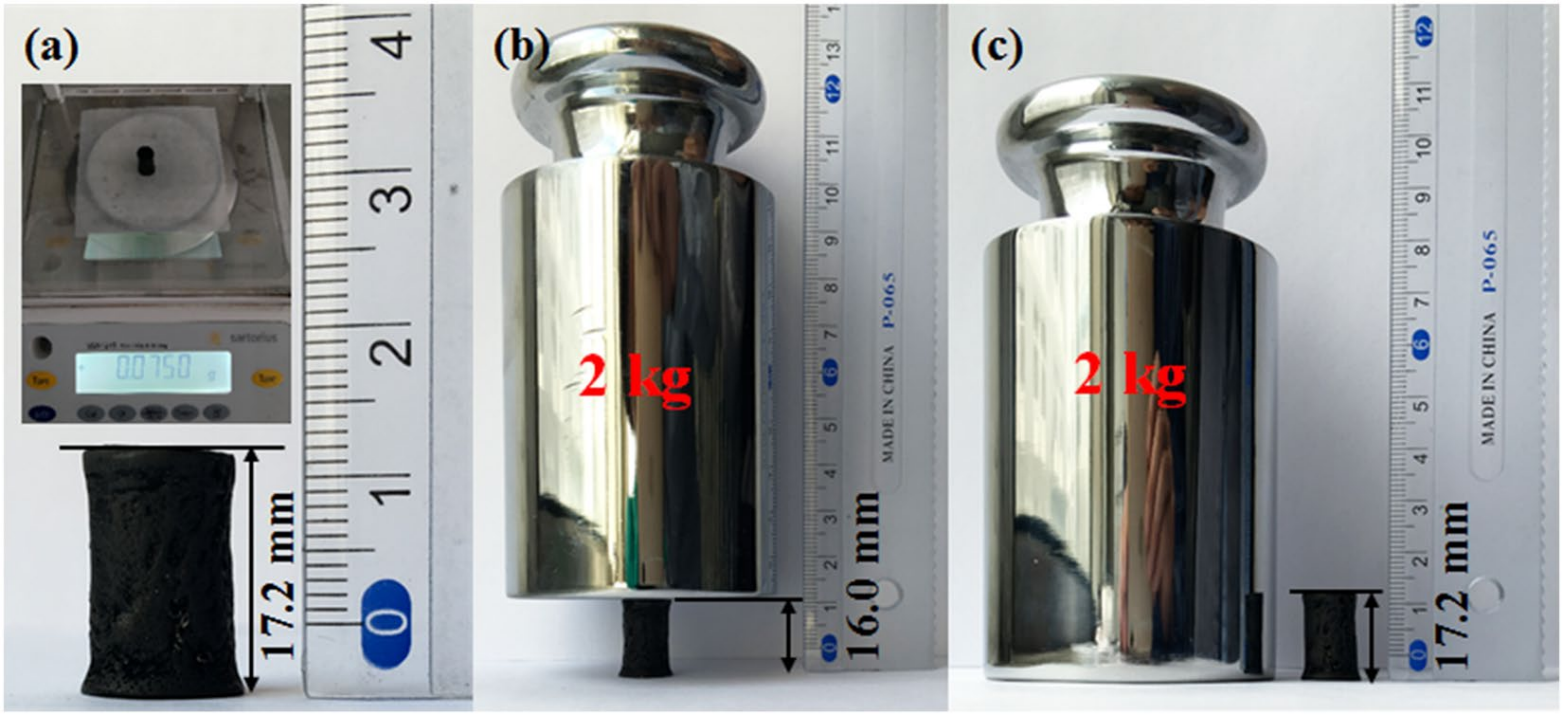
Photographs of a 75.0 mg graphene aerogel cylinder dried by supercritical ethanol supporting a 2 kg counterpoise without deformation. Digital photos of graphene aerogel in (**a**) original, (**b**) loaded, and (**c**) unloaded conditions.

Figure 5a shows the digital photos of the original GA-S5 with a bulk density of 56.2 mg∙cm^−3^ obtained from purified hydrogel dried by supercritical ethanol. A 75.0 mg graphene aerogel cylinder could support a 2 kg counterpoise, at least 26000 times their own weight (Fig. 5b). The graphene aerogel could recover to its pristine status without compressive deformation after the load was released (Fig. 5c).

**Figure 6:**
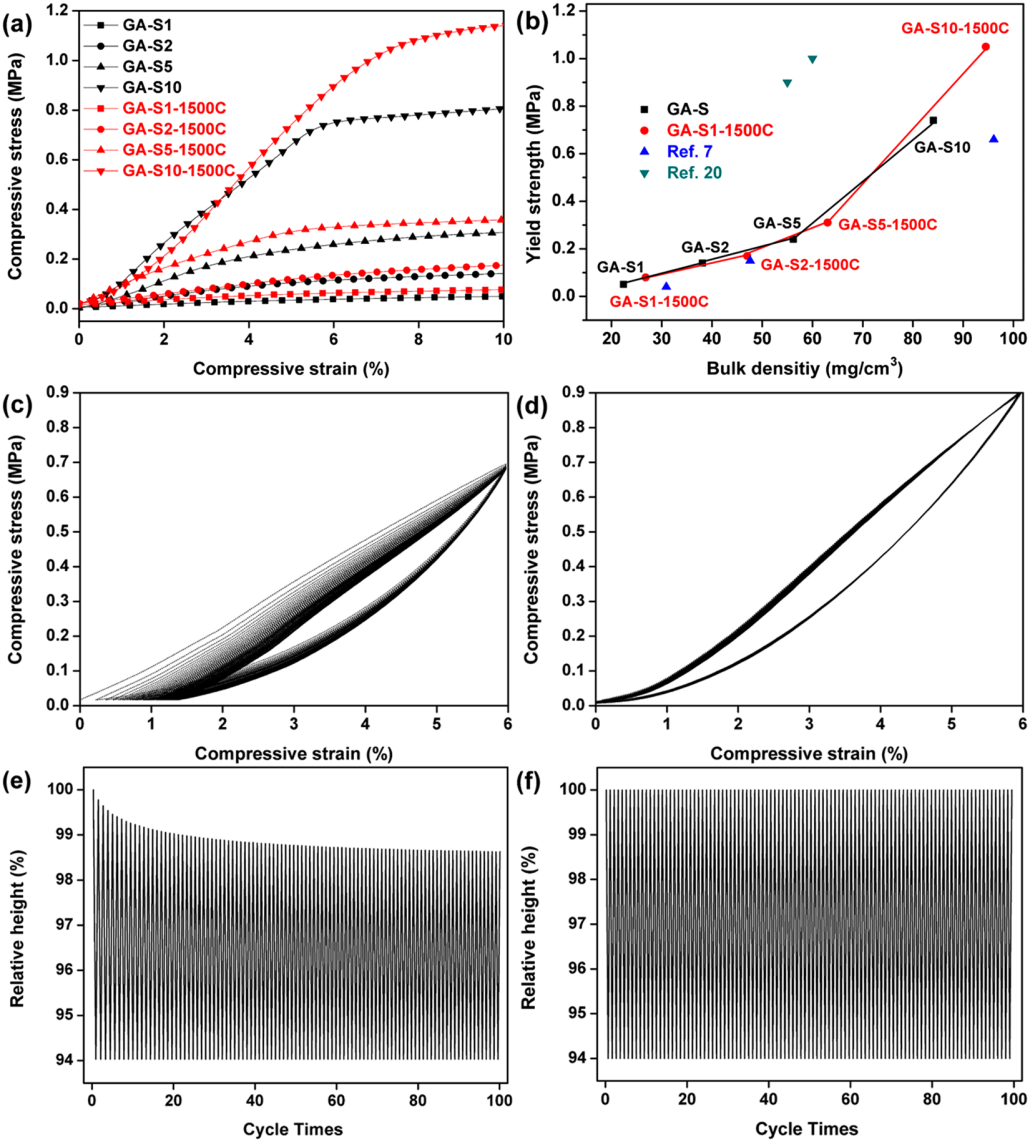
(**a**) Typical compressive stress–strain curves for GA-S (black) and GA-S-1500C (red) in the strain range of 0–10%. (**b**) The comparison of yield strength/density between GA-S, GA-S-1500C and other mechanically strong graphene aerogels. Stress–strain curves of (**c**) GA-S10 and (**d**) GA-S10-1500C upon repeated compression to 6% strain for 100 cycles. Relative heights of graphene aerogel cylinders in cyclic compression process for (**e**) GA-S10 and (**f**) GA-S10-1500C.

Due to the elastic deformation of the 3D network assembled by graphene sheets, the compressive stress–strain curves in Fig. 6a for GA-S (black curves) and GA-S-1500C (red curves) both demonstrated a linear elastic region during where the stress increases linearly with the strain. The mechanical properties of GA-S and GA-S-1500C improved in varying degrees when C_GO_ was increased from 1 mg∙mL^−1^ to 10 mg∙mL^−1^.

The bulk densities, yield strengths, and elasticity moduli of GA-S and GA-S-1500C were shown in Table S1. The yield strengths and Young’s moduli of GA-S in the elastic region were in range of 0.05–0.74 and 0.81–13.84 MPa, respectively. After annealing at 1500 °C, those of GA-S-1500C further increased to the ranges of 0.08–1.05 and 1.02–17.29 MPa, respectively. The larger crystalline domains resulted in improved stiffness of graphene sheets after the graphene aerogels were annealed at high-temperature^21^ which could account for the improvement in the yield strengths and Young moduli for GA-S-1500C. The GA-S10 and GA-S10-1500C samples had lower bulk densities and higher strengths in the linear elastic region than the supercritical CO_2_-dried graphene aerogel with a larger bulk density of 96.1 mg∙cm^−3^ as reported by Zhang *et al*.^7^ (Fig. 6b), furthermore, the elasticity moduli of GA-S and GA-S-1500C were also larger than it (6.2 MPa). The heat-treated graphene aerogels without cross-linking polymer that would weaken thermal stability had stronger mechanical properties in this work, especially the GA-S10-1500C whose yield strength (1.05 MPa) was even slightly larger than the PEI-crosslinked aerogels prepared by Huang *et al*.^20^ (Fig. 6b), but the bulk density was larger than them. GA-S10 and GA-S10-1500C could maintain their elasticities for at least 100 cycles upon repeated compression to 6% (Fig. 6c and d) with maximum yield strengths of approximately 0.7 and 0.9 MPa, respectively. The highest relative height of GA-S10 first declined gradually and then became flat along with the growth of time in the cyclic compression process (Fig. 6e). A minor unrecoverable deformation was generated in each cyclic compression, and the relative height of the sample was shortened by approximately 1.43% after 100 cycles, whereas the compressive curves of GA-S10-1500C exhibited perfect superposition after each compressive cycle, and the sample could recover to its original height. The relative height–time curve of GA-S10-1500C maintained the same in each compressive cycle (Fig. 6f), and the relative height of graphene aerogel cylinder could restore completely when the load was fully removed in the unloading process. This property made it suitable for use as pressure sensing materials. The enhanced ability of recoverable deformation of GA-S-1500C may be ascribed to the higher Young modulus and stiffness of graphene sheets after high-temperature annealing. Annealing of the graphene aerogels increased the area of hysteresis loop between the loading and unloading curves, demonstrating that GA-S-1500C dissipated more energy.

Young’s modulus and yield strength of GA-S5 (ρ = 56.2 mg∙cm^−3^) were 5.13 and 0.25 MPa, respectively, whereas those of GA-F (ρ = 48.0 mg∙cm^−3^) with the same C_GO_ were only 1.05 and 0.06 MPa, respectively (Fig. S5). The poor mechanical property is ascribed to the presence of unordered and unconnected graphene sheets that could not support the load effectively. The mechanical property indicated that the graphene aerogels dried by supercritical ethanol were rather robust, and the supercritical ethanol drying had a significant effect on the microscopic morphology and strength of the graphene aerogels.

### Thermal properties

**Figure 7:**
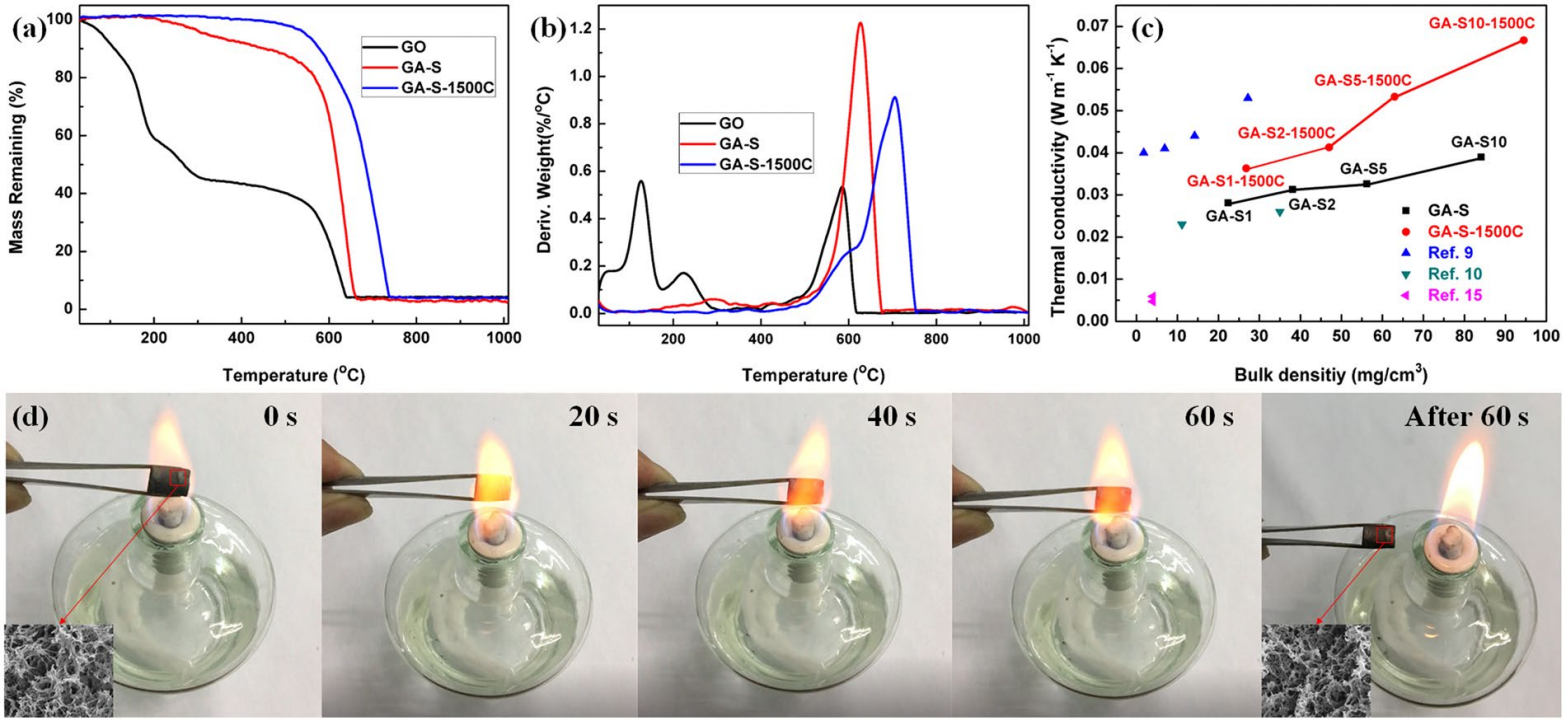
TG (**a**) and DTG (**b**) plots of GO, GA-S and GA-S-1500C in air. (**c**) Thermal conductivities of GA-S, GA-S-1500C and other low thermal conductive graphene aerogels. (**d**) Snapshots of GA-S-1500C sample in a hot flame of an alcohol burner, the insets in (**d**) were the microstructure of tested sample.

The thermal stability of GO, GA-S and GA-S-1500C in air were determined by thermogravimetric analysis. Figure 7a demonstrates that GO became more resistant to oxidation in air after it was reduced in hydrothermal process or annealed at 1500 °C for 1 h. Thermogravimetric curves indicated that the GA-S-1500C only had a weight loss of less than 6% at 500 °C, however, GO and GA-S had weight loss of approximately 60% and 15%, respectively. As seen from the DTG plots (Fig. 7b), the GO shows two obvious peaks of weight loss located at 125 °C and 585 °C, respectively. The first weight loss in the range of 80–170 °C can be attributed to the evaporation of absorbed water and the decomposition of labile oxygen functional groups^39^. The second weight loss in the range of 500–620 °C was ascribe to the combustion of graphene^39^. The main weight loss of GA-S and GA-S-1500C was occurred in the ranges of 520–680 °C and 520–750 °C, respectively. The oxidation temperature (the temperature of maximum rate of mass loss)^22^ for GA-S increased from approximately 625 °C to nearly 705 °C after annealing. This improvement in thermal stability was ascribed to the decrease in the amount of oxygenated functional groups which improved the ability of graphene aerogels to tolerate higher temperature in the air. The GA-S and GA-S-1500C samples exhibited the same tendency in thermal conductivities at room temperature, which both improved with the increase of bulk density (Fig. 7c). The thermal conductivities of GA-S were in range of 0.0281–0.0390 W∙m^−1^∙K^−1^. After annealing at 1500 °C, the thermal conductivities of GA-S-1500C had a slightly increase to the range of 0.0363–0.0667 W∙m^−1^∙K^−1^. This enhancement in thermal conductivity with the increase of bulk density could be attributed to a more connected network of graphene aerogels^40^ (shown in Fig. 2). In addition, the higher density of graphene sheets and the increased overlap between graphene sheets reduced the resistivity pathways for phonon transport through the aerogels^41^ that contributed to the improved thermal conductivity. After high-temperature annealing, the thermal conductivities of all GA-S-1500C samples were higher than those of pure GA-S. Except at the high densities, the elimination of oxygen-containing functional group and healing of defects in graphene sheets could promote the transmission of heat and improve the thermal conductivity of GA-S-1500C^40^. Although the bulk densities of GA-S (22.4–84.1 mg∙cm^−3^) were much higher than those of the graphene aerogels prepared by Tang *et al*.^9^ (1.8–27.2 mg∙cm^−3^), the thermal conductivities (0.0281–0.0390 W∙m^−1^·K^−1^, 295.5 K) for GA-S shown in Fig. 7c were lower than those of the latter (0.040–0.053 W∙m^−1^∙K^−1^). This result could be ascribed to the finer pore structure of our graphene aerogels. The thermal conductivity of graphene aerogels can be as low as <0.01 W∙m^−1^·K^−1^, but bulk density and mechanical property was very low^15^. To indicate the thermal stability of graphene aerogels more visually, the GA-S-1500C graphene aerogel was tested in a hot flame of an alcohol burner (Fig. 7d). When exposed to the hot flame for 60 s, the graphene aerogel cannot be burned. It remained the unbroken shape, original size, inherent 3D porous structure, even though we repeated this procedure several times. The excellent fire resistance mainly be ascribed to the flame temperature of an alcohol burner was lower than the combustion temperature of graphene aerogels (provided in thermogravimetric curves of Fig. 7a).

### Electrical properties

**Figure 8:**
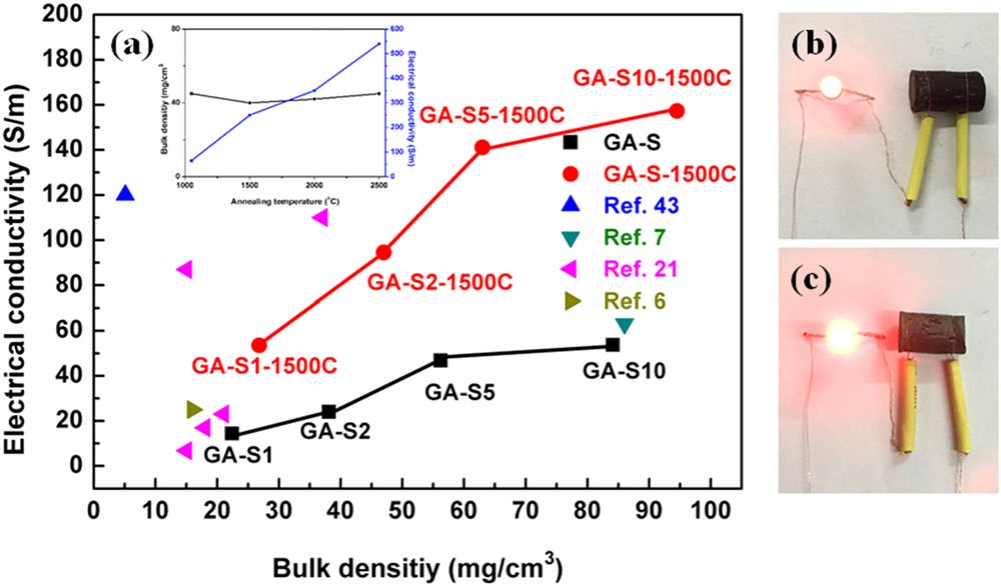
Electrical conductivities (**a**) of GA-S, GA-S-1500C and other high electric conductive graphene aerogels, the inset in (**a**) was the bulk density and electrical conductivity of graphene aerogels vs annealing temperature in ref. 22. A circuit constructed with the GA-S (**b**), GA-S-1500C (**c**) cylindrical sample.

The bulk electrical conductivities of graphene aerogels measured by four-probe method were shown in Fig. 8a. The electrical conductivities of GA-S and GA-S-1500C samples both increased with bulk densities because the sample with higher density is easier to transfer electrons^42^. The bulk electrical conductivities of GA-S increased from 14.4 to 53.7 S/m with the increase of bulk density. After annealing at 1500 °C, those of GA-S-1500C further increased to the ranges of 53.5–157.3 S/m, respectively. Compared to GA-S samples, a significant improvement in electrical conductivity of high-temperature annealed GA-S-1500C samples was observed. Except higher density, restored conjugated domains and diminished oxygenated functionalities^23^ (as confirmed by the XRD and XPS results) also enhanced the electrical conductivity of GA-S-1500C samples. Although, the GA-S10-1500C has higher electrical conductivity than recent reported graphene aerogels^6, 7, 21, 43^, but lower than the highly crystalline graphene aerogels (annealing temperature ≥1500 °C, inset in Fig. 8a)^22^. A LED lamp can be illumined under 3 V circuit when connected with a graphene aerogel cylinder, and the lamp connected with a GA-S-1500C samples (Fig. 8b) was obviously brighter than the one connected with GA-S samples (Fig. 8c).

## Conclusion

In summary, we prepared mechanically strong graphene aerogels with low thermal conductivities, high thermal stability and electrical conductivities by hydrothermal reduction of GO dispersions and supercritical ethanol drying. The yield strengths and Young’s moduli of GA-S were in range of 0.05–0.75 and 0.81–13.84 MPa, respectively. A 75.0 mg graphene aerogel cylinder with a bulk density of 56.2 mg∙cm^−3^ could support at least 26000 times its own weight. The thermal conductivities and electrical conductivities of GA-S were in range of 0.0281–0.0390 W∙m^−1^·K^−1^ and 14.4–53.7 S/m, respectively. Annealing at 1500 °C resulted in a further improvement in the yield strengths and Young’s moduli of graphene aerogels to 0.08–1.05 MPa and 1.02–17.29 MPa, respectively. The GA-S10-1500C could maintain its elasticity for at least 100 cycles upon repeated compression to 6% accompanied by the maximum yield strength of 0.9 MPa without unrecoverable deformation. With high-temperature annealing, the oxidation temperature of graphene aerogels in air increased from approximately 625 °C to almost 705 °C, and the thermal conductivity and electrical conductivities of graphene aerogels increased to the ranges of 0.0363–0.0667 W∙m^−1^·K^−1^ and 53.5–157.3 S/m, respectively. The large BET surface areas, together with strong mechanical properties, low thermal conductivities, high thermal stability and electrical conductivities made these graphene aerogels feasible candidates for use in a number of fields covering from batteries to pressure sensors, electrodes, lightweight conductor and insulation materials.

## Methods

### Materials

Graphite flakes (325 mesh, >99%, USA) were used to prepare GO through a modified Hummers’ method. Sodium nitrate (NaNO_3_), hydrogen peroxide (H_2_O_2_, 20 wt.%), and sodium hydrogen sulfite (NaHSO_3_) were purchased from Tianjin Bodi Chemical Co., LTD. (Tianjin, China). Potassium permanganate (KMnO_4_), concentrated sulfuric acid (H_2_SO_4_, 98 wt.%), and hydrochloric acid (HCl, 30 wt.%) were obtained from local suppliers (Harbin, China).

### Synthesis of the aerogels

GO was prepared from natural graphite using the improved Hummers method^25^. The 1.3 nm average height of the GO sheets indicated that the synthesized GO sheets have a thickness of 1 to 2 atomic layers (Fig. S1a and b). The larger volume shrinkage and high cross-linking degree of GO sols under the action of the mild reductant NaHSO_3_^17^ have led to strong graphene gels. The graphene gels are dried by supercritical ethanol to maintain the fine framework of the gels during solvent removal, and then mechanically strong graphene aerogels are obtained. The graphene aerogels are annealed at high temperature to further improve thermal stability, electrical conductivity and mechanical properties. The synthesis of the mechanically strong graphene aerogels with cylindrical morphology is illustrated in Fig. 1. Graphene aerogels with different densities were prepared by adding 0.11 g of NaHSO_3_ into 30 mL of 1, 2, 5, and 10 mg∙mL^−1^ GO aqueous dispersion with vigorous magnetic stirring until completely dissolved. Then, the homogeneous GO aqueous dispersion was sealed in Teflon-lined autoclaves and maintained at 180 °C for 12 h to form graphene-based three-dimensional (3D) hydrogels. The obtained hydrogels were first purified in a large amount of ethanol for at least 1 week and then dried using supercritical ethanol at 270 °C with a pressure of 8 MPa to obtain the graphene aerogels. The resulting samples were referred to as GA-S. The obtained GA-S samples were then heat treated at 1500 °C for 1 h under N_2_ atmosphere. The resulting samples were labeled as GA-S-1500C. The GA-S and GA-S-1500C prepared with various concentrations of GO (C_GO_) were denoted as GA-Sx and GA-Sx-1500C, where x is the concentration of GO dispersion in milligram per milliliter. In addition, we investigated the effect of the drying process by freeze-drying graphene aerogels with GO aqueous dispersion of 5 mg∙mL^−1^ using the same method as that for GA-S, but deionized water, instead of ethanol, was used to purify the hydrogels. The resulting sample were denoted as GA-F.

### Characterization and measurement

Atomic force microscopy (AFM, Dimension Icon, Bruker, Germany) in tapping mode at a scan rate of 0.999 Hz was performed to obtain AFM images of the GO sheets. The bulk densities of the resulting aerogels have been calculated by the mass of the graphene aerogel divided by its volume. The microscopic morphology of the graphene aerogel was investigated via a scanning electron microscope (FEI Helios Nanolab 600i, USA). Nitrogen adsorption–desorption measurements were performed using a Micromeritics 2020 ASAP instrument at 77 K. Surface areas were calculated using the BET (Brunauer-Emmett-Teller) method, and the pore size distribution was calculated by the Barrete-Joynere-Halenda (BJH) method. XPS (X-ray photoelectron spectroscopy) analysis of GO, GA-F, GA-S, and GA-S-1500C was performed on an ESCALAB 250Xi photoelectron spectrometer (ThermoFisher Scientific, Waltham, Massachusetts, USA). X-ray diffraction (XRD) patterns of GO, graphene aerogels, and natural graphite were analyzed by a D/max-RB XRD (Rigaku Corporation, Tokyo, Japan). Raman spectra were measured by irradiation with laser light at 532 nm in a Renishaw spectrometer. Compression tests were conducted using an electronic universal material testing machine (Instron-5569, USA) with loading capacity of 1000 N at a constant loading speed of 2 mm/min. Thermal conductivity was measured by hot wire technique using a thermal conductivity measuring instrument (TC 3000E, Xi’an Xiatech Electronic Co., Ltd., China). Thermal stability of the graphene aerogels was investigated by thermogravimetric analysis (TGA, TA Instruments TGA 2050, USA) with a heating rate of 15 °C∙min^−1^ under air atmosphere. The bulk electrical conductivities of cylindrical graphene aerogels were measured by four-probe method with metal electrodes attached to the ends of samples.

## Electronic supplementary material

Supplementary Information
