# Supplementary material for: Enhanced mechanical, thermal, and electric properties of graphene aerogels via supercritical ethanol drying and high-temperature thermal reduction
Source: Sci Rep. 2017 May 3;7:1439. doi: 10.1038/s41598-017-01601-x (PMC5431159; doi:10.1038/s41598-017-01601-x)
Supplement: Supplementary file 1 — Supplementary Information [file 41598_2017_1601_MOESM1_ESM.pdf]

Supplementary Information of

Enhanced mechanical, thermal, and electric properties of graphene aerogels via supercritical ethanol drying and high-temperature thermal reduction

Yehong Cheng[1], Shanbao Zhou[1], Ping Hu[1], Guangdong Zhao[1], Yongxia Li[1], Xinghong Zhang[1]*, Wenbo Han[1]*

[1]National Key Laboratory of Science and Technology on Advanced Composites in Special Environments, Harbin Institute of Technology, Harbin 150001, People's Republic of China

* Corresponding author e-mail address: zhangxh@hit.edu.cn, wbhan@hit.edu.cn

Supplementary Figures:

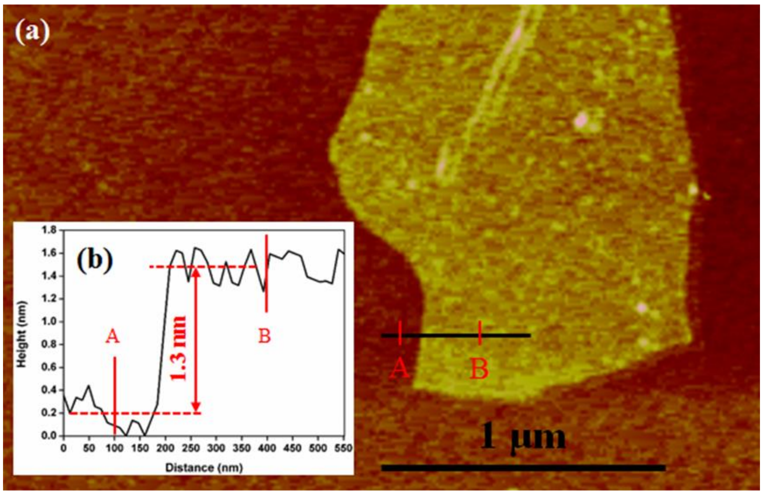

Fig. S1 (a) Atomic force micrographs of graphene oxide (GO) sheets deposited on a freshly cleaved silicon slice surface and (b) the height profile of a GO sheet

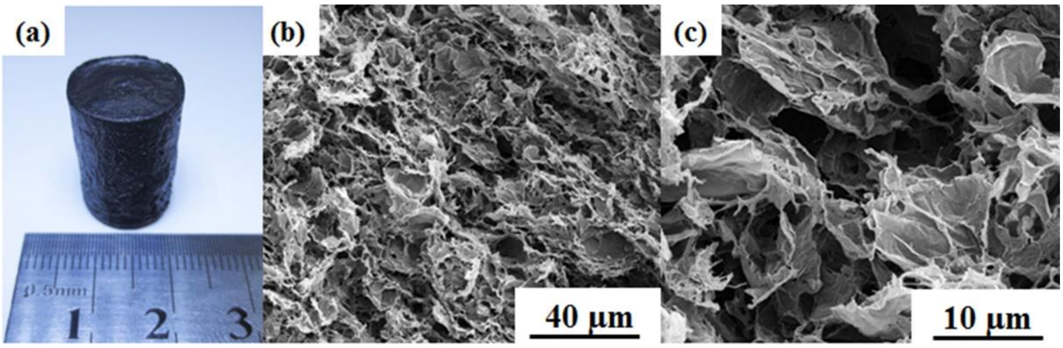

Fig. S2 (a) Digital photographs and (b and c) microscopic morphology of the GA-F prepared by hydrothermal reduction of 5 mg·mL^{-1} GO

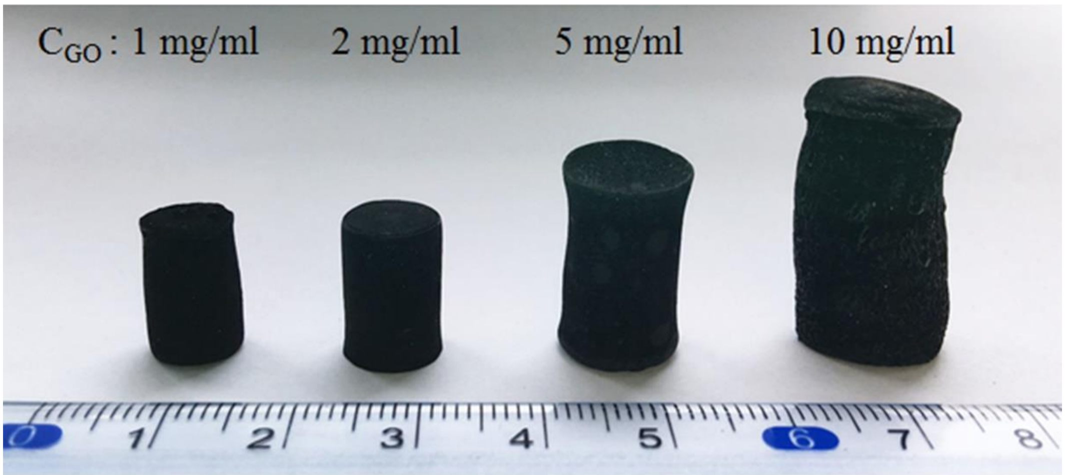

Fig. S3 Digital photographs of GA-S prepared by 1, 2, 5, and 10 mg·mL^{-1} GO aqueous dispersion

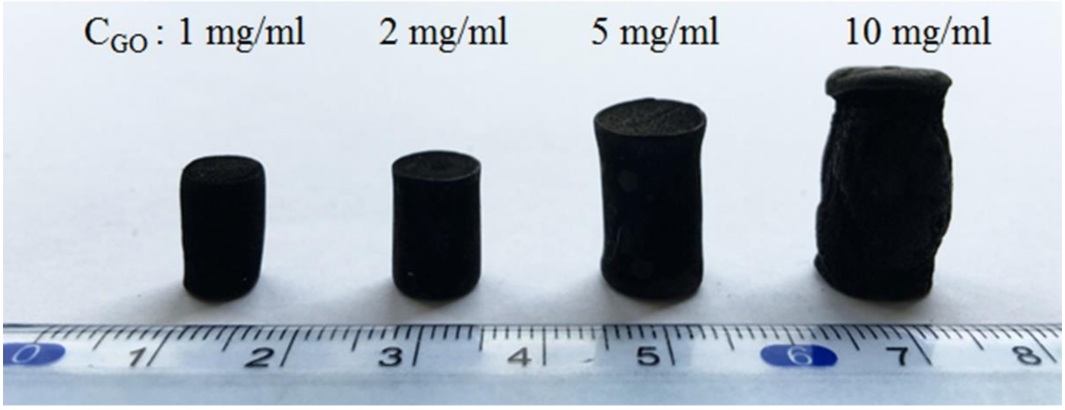

Fig. S4. Digital photographs of GA-S-1500C prepared by 1, 2, 5, and 10 mg·mL^{-1} GO aqueous dispersion

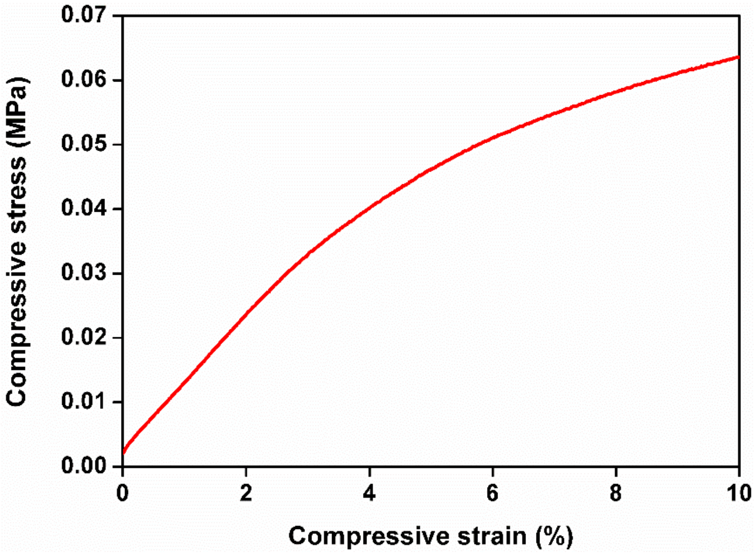

Fig. S5. Mechanical property for GA-F prepared by hydrothermal reduction of 5 mg·mL^{-1} GO dispersion

Table S1 The bulk densities (ρ), yield strengths (σ), elasticity moduli (E), Brunauer-Emmett-Teller (BET) surface areas (S_{BET}), Barrete-Joynere-Halenda (BJH) method cumulative desorption surface area (S_{BJH}), pore volumes, and average pore diameters of GA-S and GA-S-1500C with different starting GO concentrations.

Composition	ρ ($mg \cdot cm^{-3}$)	σ (MPa)	E (MPa)	S_{BET} (m^2/g)	S_{BJH} (m^2/g)	Pore volume (cm^3/g)	Pore diameter (nm)
GA-S1	22.4	0.05	0.81	593.6	1179	4.487	3.822
GA-S2	38.1	0.14	1.70	602.8	871	2.848	3.841
GA-S5	56.2	0.24	5.83	502.1	860	2.369	3.834
GA-S10	84.1	0.74	13.84	435.6	516	2.153	3.817
GA-S1-1500C	26.8	0.08	1.02	336.5	510	2.716	3.791
GA-S2-1500C	47.0	0.17	2.04	384.0	770	8.305	3.776
GA-S5-1500C	63.0	0.31	6.96	423.1	674	5.733	3.771
GA-S10-1500C	94.5	1.05	17.29	440.8	905	7.125	3.784
